# Antimicrobial and Histological Data Effect of *Silybum marianum* and *Suaeda vermiculata* Against Doxorubicin Induced Toxicity in Male Rats

**DOI:** 10.31557/APJCP.2021.22.6.1761

**Published:** 2021-06

**Authors:** Sarah Samir Othman, Safaa Mohamed Ali

**Affiliations:** 1 *Department of Pharmaceutical Bioproducts Research, Genetic Engineering and Biotechnology Research Institute, City of Scientific Research and Technological Applications, New Borg El-Arab City 21934, Alexandria, Egypt. *; 2 *Department of Nucleic Acid Research, Genetic Engineering and Biotechnology Research Institute, City of Scientific Research and Technological Applications, New Borg El-Arab City 21934, Alexandria, Egypt. *

**Keywords:** Silybum marianum, Suaeda vermiculata, hepatoprotective, doxorubicin, antimicrobial

## Abstract

**Objective:**

*Silybum marianum* and *Suaeda vermiculata* are popular plants wealthy in cancer prevention agents. There is no enough research on both plants since they are not available in many places. They are widely spread in Egypt.

**Methods:**

This research was performed to estimate their antimicrobial effect as well as their hepatoprotective effect against strong drugs inducing oxidative stress such as doxorubicin which may be a chemotherapeutic operator utilized to treat different sorts of cancer and demonstrated to be hepatotoxic medicate. Six bunches of male Wistar rats were utilized (control, S*ilybum marianum extricate, Suaeda vermiculata extricate, doxorubicin, Silybum marianum extricate* additionally doxorubicin and *Suaeda vermiculata* extricate additionally doxorubicin).

**Results:**

Our data confirmed the effective antimicrobial effect of both plants and also the hepatoprotective effect against oxidative damage. Both plants are highly recommended as natural supplements by patients treated by different drugs inducing oxidative stress whereas; Milk thistle was proved to be stronger hepatoprotective herb.

## Introduction

Doxorubicin could be a chemotherapeutic sedate utilized to treat various sorts of cancer counting breast cancer and intense lymphocytic leukemia. It is infused intravenously. This drug causes numerous side effects including hair loss, bone marrow suppression as well as proved to be a cardio toxic drug (The American Society of Health-System Pharmacists, 2017). The main mechanism of action of this medication is by causing oxidative stress (Mai et al., 2016). In spite of the suggestions of the World Health Organization (WHO), that states that an extensive numeral of curative plants are utilized by the common populace without any logical bolster, making the utilize silly and possibly lethal when within the nearness of comorbidities (Villas Boas et al., 2018). Pharmaceutical research focuses on the development of promising as well as safer synthetic/natural drugs against ulcer. Herbal drugs are currently regarded as promising choice of drug materials in the quest of new medications for numerous diseases (Santin et al., 2010). The mind blowing increment within the request of drugs of plant beginning leads to over abuse of restorative plants which eventually lead to the shortage and endangerment of numerous profitable plant species (Alshryda et al., 2013). Ethnopharmacology has energized considers of the restorative and wholesome potential of endemic botanical species in particular locales and broadly utilized by the populace at these locales. Hence, ethnopharmacologists and doctors are inquisitive about the arbitrary determination of restorative plants, information collection and ponders that point at a profound understanding of conventional restorative epistemology (Dragos and Gilca, 2018). *Silybum marianum* (Milk thistle) was proved to cure numerous diseases including liver diseases and as a natural source of strong antioxidants (Othman et al., 2020). *Suaeda vermiculata* mainly exits in Africa and the Middle East (African Plant Database, 2019) and it is rich in flavonoids, phenolics and alkaloids which indicate its strong activities. It was also proved to have antimicrobial activity. *Suaeda vermiculata* was moreover demonstrated to be a novel source for the investigation of unused antioxidant and antimicrobial specialists that are possibly esteemed for nourishment and biomedical applications (Al-Tohamy et al., 2018). Our inquire about pointed to utilize both plants as characteristic sources of cancer prevention agents to overcome the oxidative stretch initiated by doxorubicin (chemotherapeutic medicine). Their antimicrobial exercises were moreover estimated.

## Materials and Methods

*Determination of antimicrobial effect of Silybum marianum and Suaeda vermiculata*

AST used as antimicrobial detection of resources against microbes. Susceptibility tests are important in the investigational analysis if the microbe is suspected of being antimicrobial resistance agents (Anon, 2003; Aureli et al., 2003; Benli et al., 2007; Panda et al., 2009). *Escherichia coli* (garm negative bacteria), *Bacillus *(gram positive bacteria), *Candida albican* (yeast) and *Asperigillus niger* (fungi) were used for detection of antimicrobial studies of the diverse plant extract. All microorganism were inoculated into the inoculated medium (LB liquid medium for bacteria and potato dextrose for fungi) under 220 rpm and (37°C for bacteria and 30 °C for fungi), until the OD value at around 600 nm was about 0.6. The bacterial solution was diluted at a volume ratio of 1:1,000. The plant extricates and ampicillin (0.01 gm) was connected to the microbial arrangement 3 mL, and hatched for 24 h at 220 rpm, 37° C for microscopic organisms. Brooding of Asperigillus niger for 5 days at 30°C .The OD esteem at 600 nm was measured. Information from this try was ordinarily displayed as rate of restraint to show the levels of action.

*Chemicals*

Doxorubicin was acquired from Sigma Chemical Company; St Louis, Moment, USA and was utilized concurring to the past work of Mello et aL., (2017).

*Preparation of plant extract*

100 gm wet plant were dissolved in 100 ml distilled water, they were boiled for 5 minutes then homogenized, filtered and lyophilized. Doses were prepared in which 1gm was dissolved in each 10 ml. 

*Experimental animals*

Thirty Male Wistar rats were utilized having weights 150–180 g and were kept on basal slim down and tap water which were given advertisement libitum and kept beneath standard conditions which acclimated to the National Institutes of Health (NIH) rules. After 14 days of acclimation, creatures were partitioned into 6 bunches 5 rats in each, control, administrated *Silybum marianum* extricate (100 mg/ml/Kg, orally), given *Suaeda vermiculata* extricate (100 mg/ml/Kg, orally), treated with doxorubicin (15 mg/kg BW, ip), *Silybum marianum* extricate furthermore doxorubicin and *Suaeda vermiculata* extricate also doxorubicin individually. The explore took put in 14 days, dosages of the extricate were given day by day but doxorubicin was as it were given on 14^th^ day.

*Histopathological examination*

Following sacrification, the liver specimens of the rats of all groups were collected, fixed in 10% neutral buffered Formalin solution. 24 hrs afterward, taking after steps of lack of hydration in climbing grades of ethanol, cleared in xylene and after that inserted in paraffin wax. For the histopathologic appraisal, tissue portions (3-5 Microns thick) were cut and recolored with hematoxylin and eosin (H&E) (Ban croft and Stevens, 1996) and subjected to the light microscopy.

## Results

*Antimicrobial studies of Silybum marianum and Suaeda vermiculata extracts*

The results reflect the inhibitory power of a compound only for the time specified. The micro-organism is also challenged in the descriptive study, but regular sampling is performed to determine improvements in the viable number of cells over time. The test was directed to determine extracts end product on microbial growth. Relative Viability of the different microbes after 24 h incubating with L and S extracts and Ampicillin were determine and comparing with blank (untreated microbe) as presented in fig E. The excellent antibacterial properties of *Silybum marianum* and *Suaeda vermiculata* extracts are more effective than ampicillin. 

*Histopathologic findings*

*Changes in the livers of all groups (* Figures 2 *-* 7 *)*

The light microscopy for the livers of the control group showed no histopathologic changes (Figure 2), while the liver of the rats treated with Doxorubicin showed hepatocytic degeneration and necrosis with mononuclear cell infiltration, especially at the necrotic areas, dilation and congestion of central veins (Figure 3). The livers in the group received only the *Silybum marianum* extract showed changes of active defensive effects in form of voluminous hepatocytic cytoplasm with activation of the Kupffer cells, defensive cells of the hepatic tissue (Figure 4). The livers in the group received the *Silybum marianum* extract + doxorubicin showed less vascular changes of congestion of central veins and sinusoids, while the hepatic cells not suffered necrosis but only affected with degeneration in form of fatty change (Figure 5). The group administered with only *Suaeda vermiculata* extract showed some hepatic changes of mild dilation and congestion of the central veins, while the hepatic cells looked normal (Figure 6). After double administration of *Suaeda vermiculata* extract with Doxorubicin less ameliorating effect was noticed, where the congestion appeared more progressed and zones of hemorrhages and ruddy blood cell extravasations showed up to scattering the hepatic tissue (Figure 7).

## Discussion

Chemotherapeutic operators cause oxidative stress in patients which is the most reason of their side impacts (Yokoyama et al., 2017). *Silymarin* (active extract from milk thistle seeds) (Taleb et al., 2018) and *Suaeda vermiculata* are proved to have effective antioxidant effect and protection against oxidative stress (Elsharabasy et al., 2019). Functions for metabolic and elimination occur in the liver and kidney. During this process, liver and kidneys may suffer damage due to ingestion or toxic metabolites formation leading to organ loss and even death (Sumera et al., 2020). The liver is among the most crucially important organs of the body to maintain and regulate homeostasis. Liver failure, known as hepatotoxicity, is mainly caused by chemical agents such as environmental toxins or pharmacotherapy (Bjornsson, 2016). Drug-induced hepatotoxicity in definite, is the most reason of with drawl of endorsed drugs from the advertise (Pugh et al., 2009). Silymarin, derived from *Silybum marianum* seeds, was among the herbal extracts which have been shown to have a protective effect on hepatotoxicity caused by drugs. Protection of hepatocytes against various toxins has been reported (Wahkheong et al., 2017). It has been used clinically either alone or as a major component of various pharmaceutical preparations as a hepatoprotective and antiinflammatory agent (Avci et al., 2016). The detected hepatotoxic changes of doxorubicin were in form of hepatocytic degeneration and necrosis, mononuclear cell infiltration, dilation and vascular congestion and hemorrhages. On the same time, as done in the present study, the ameliorating effect of *Silybum marianum* extract was found to be somewhat obvious than in case of *Suaeda vermiculata* extract. Our histological results are consistent with Rajasekaran and Periyasamym (Rajasekaran and Periyasamy, 2010). Hahn et al., (1968) clarified the anti-hepatotoxic component of Silymarin is related with its stabilizing impact on cytoplasmic layers. It is also protective against alcoholic-induced liver damage (Jia at al., 2013), diethylnitrosamine induced liver disease (Pappachan et al., 2014), hepatitis C virus and carbon tetrachloride-induced oxidative disorders (Voroneanu et al., 2016). Merrell and Cherrington, (2011) illustrated Silymarin alleviates alteration o.f histological structure as they stated that hepatic histological changes are direct indication of the degree of liver injury. They expressed that Silymarin inhibits hepatocyte apoptosis and inhibiting lipid peroxidation, the inflammatory response. They affirmed that the hepatoprotective impacts of Silymarin were prove by the nonappearance of cellular rot and aggravation within the liver segment, especially in rats treated with center and high-dose Silymarin. It has been detailed that Silymarin plays a crucial part as an antioxidant by rummaging oxygen free radicals and diminishing lipid peroxidation to quicken hepatocyte recovery with negligible unfavorable activities (Feng et al., 2016). Our protective findings of Suaeda extract agree with Elsharabasy et al., (2019) who found that Suaeda extract had significantly reduced the elevated levels of ALT, AST, and ALP explainig that these biochemical restorations could be due to the extract ability to inhibit the cytochrome P450 or/and ability to promote the PCM glucuronidation. They added that that *Suaeda* extract contain cysteine and Methionine with excessive amount which considered as -SH donner for GSH synthesis. They also proved that this extract has hepatoprotective effect. They explained its antioxidant activity due to the presence of high amount of α linolenic and linoleic fatty acid.

In conclusion, *Silybum marianum* and *Suaeda vermiculata* are two strong herbs that are rich in antioxidants and antimicrobial agents. They are beneficial supplements to patients treated with various drugs proved to have oxidative and inflammatory effects.

**Figure 1:**
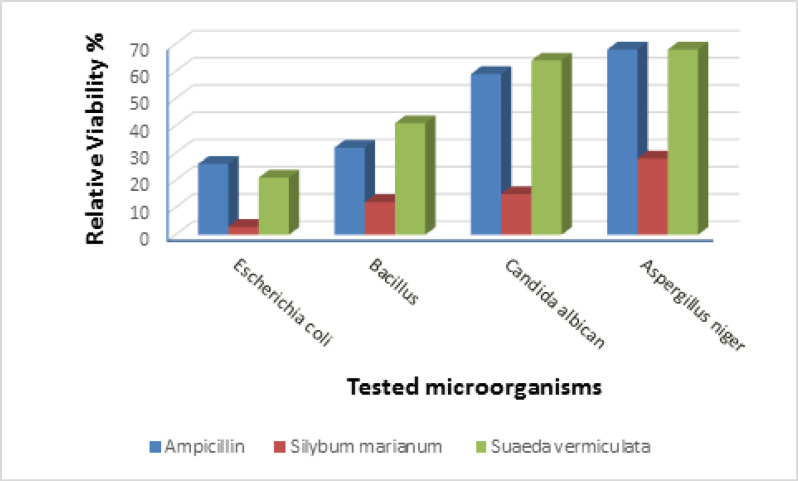
Diagram Show Relative Viability after 24 h Incubation with Different Antimicrobial Agents (Plants Extracts) against Different Microorganism Categories

**Figure 2:**
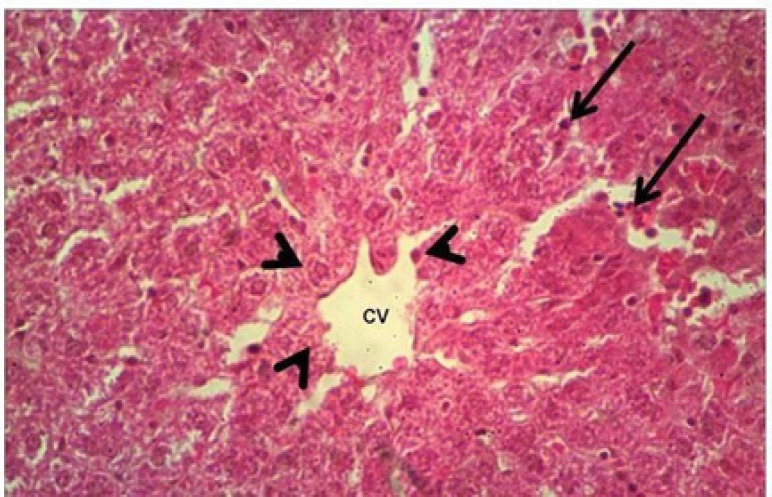
Control Group: normal hepatic tissue with hepatocytic cords (arrow head) surrounding to a clear non congested central vein (CV), with presence of some few Kupffer cells (arrows). HandE, X400

**Figure 3:**
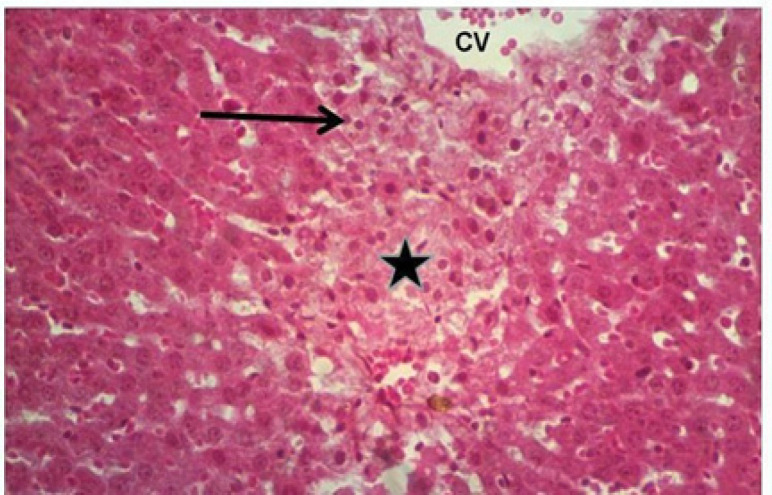
Doxorubicin Group: area of necrosis (asterisk) with excess mononuclear cell infiltration (arrow) near to a congested central vein (CV). HandE, X400

**Figure 4:**
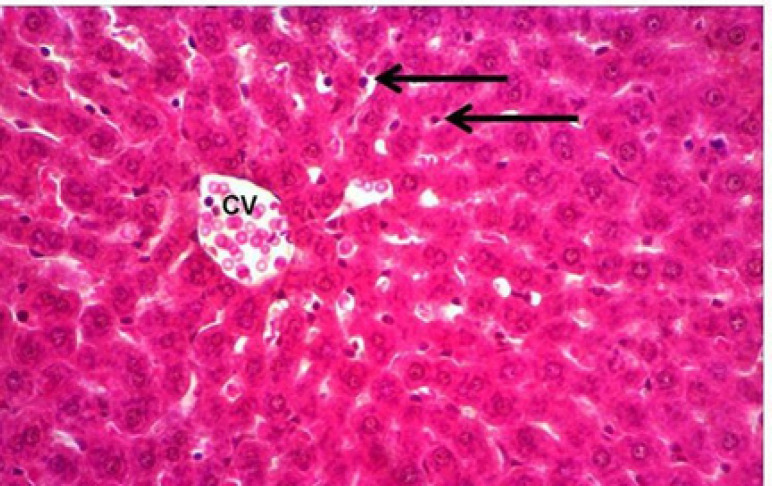
*Silybum marianum* Extract Group: most of the hepatic cells with voluminous cytoplasm arranged around a congested central vein (CV) and separated by numerous and active Kupffer cells (arrows). HandE, X400

**Figure 5:**
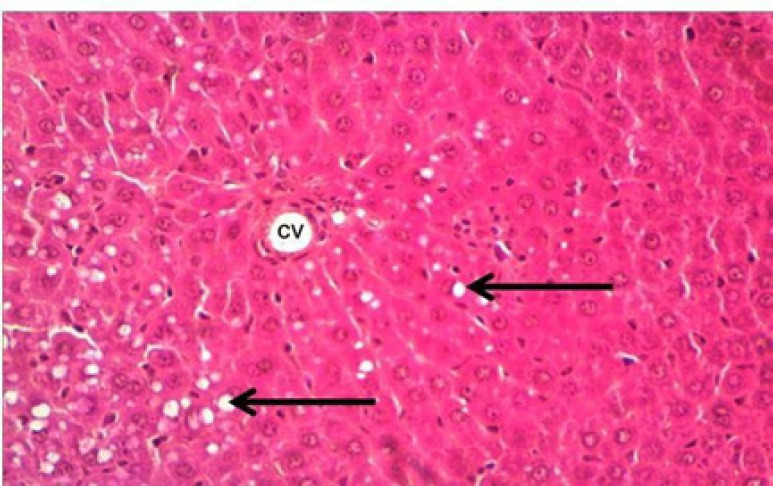
*Silybum marianum* Extract +doxorubicin Group: central vein (CV) with a surrounding cords of hepatic cells suffered sharp edged vacuolation of fatty change (arrow). HandE, X400

**Figure 6:**
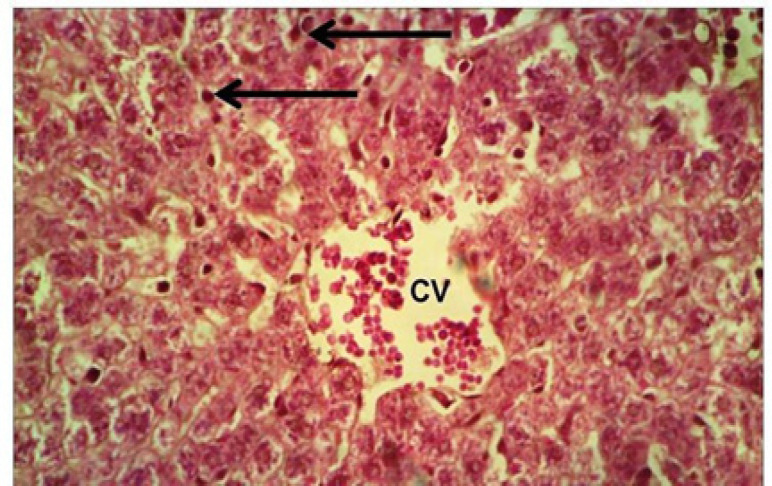
*Suaeda vermiculata* Extract Group: dilated and congested central vein (CV) with excess of active Kupffer cells and few lymphocytic infiltration (arrows). HandE, X400

**Figure 7:**
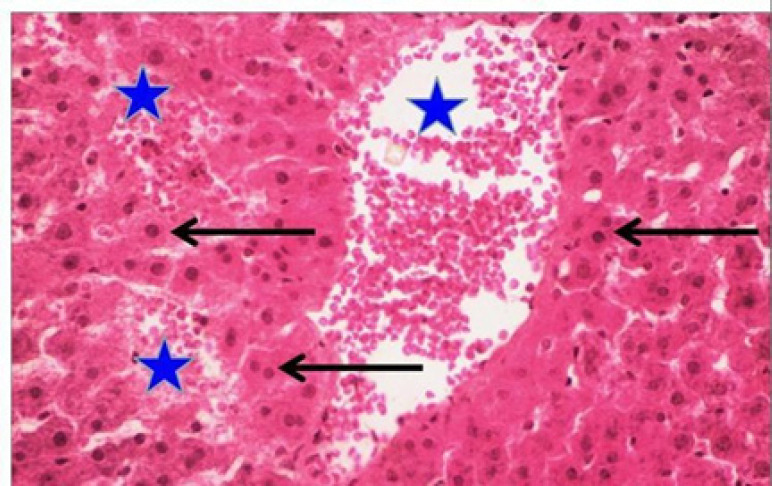
Suaeda Vermiculata Extract +Doxorubicin Group: hepatic cells appeared degenerated and dispersed (arrow) by bloody areas of free extravasated erythrocytes (asterisks). HandE, X400

## Author Contribution Statement

Sarah Samir Othman and Safaa Mohamed Ali participated in planning the study and developing the experimental design. SSO participated design and coordination of the animal study, biochemistry study, uptake assays and participated in the statistical analysis. SMA carried out of plant extract, antimicrobial activity and carried out the per-formed the DAT autoradiography. All authors read and approved the final manuscript.
